# Floating thrombus in the right heart associated with pulmonary embolism: The role of echocardiography

**DOI:** 10.12669/pjms.311.6219

**Published:** 2015

**Authors:** Kashif Naeem

**Affiliations:** Kashif Naeem, MRCP(UK), Department of Cardiology, Al Baraha Hospital, Dubai, UAE.

**Keywords:** Free-floating thrombus, Pulmonary embolism, Echocardiography

## Abstract

Free-floating right heart thrombi are rare and usually represent travelling clots from venous system to the lung. Almost exclusively, they are associated with pulmonary embolism. Despite associated high mortality, they are frequently under-diagnosed. We report a case of bilateral pulmonary embolism which was found to have a free-floating right atrial thrombus on echocardiography. The case, therefore, highlights the importance of echocardiography as a key examination in this setting. It can be performed at bedside to directly visualize the thrombi, assess and monitor right ventricular (RV) function, and help in making treatment decisions.

## INTRODUCTION

A thrombus in the right heart in the absence of atrial fibrillation, structural heart disease or catheters in-situ is rare. It usually represents a travelling clot from the venous system to the lung. In view of the reported high mortality, it constitutes a medical emergency and requires immediate treatment. We hereby, report a case where a free-floating right heart thrombus was detected in a patient with bilateral pulmonary embolism, and review the related literature.

## CASE DESCRIPTION

**Fig.1:**
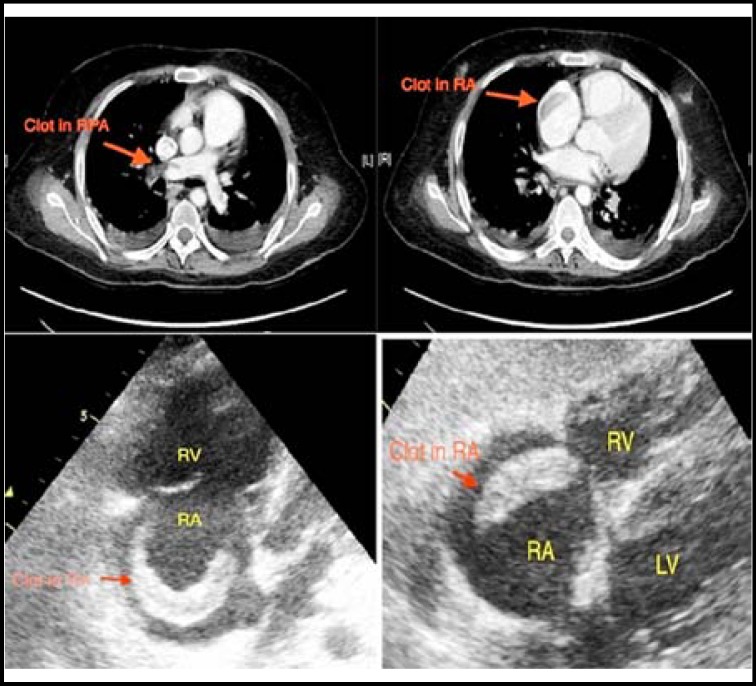
Computed tomography of the chest (top panel) showing filling defects in the right pulmonary artery (top left) and right atrium (top right) suggesting thrombi. The bottom panel shows 2-D echocardiography showing ‘snake-like’ thrombi in the right atrium (apical and subcostal views).

A 56-year-old man was electively admitted for investigation of his abdominal pains. He had surgery for traumatic fracture of his right leg three weeks ago and was discharged without any complications. He did not have any other major co-morbidity and was not taking any regular medications. Few days into the hospital stay, he suddenly became short of breath and unwell with severe hypoxemia, hypotension and tachycardia. His peripheral oxygen saturations dropped to 85% on room air. His systolic blood pressure was 60 mmHg with heart rate of 120 per minute. After initial stabilization, urgent computerised tomography scan of the chest was performed that revealed filling defects in the distal main pulmonary artery extending bilaterally up to the interlobar and its segmental branches, consistent with thrombi (Fig.1, top left). It also revealed dilated pulmonary trunk (24 mm) with a filling defect in the right atrium (Fig.1, top right). A trans-thoracic echocardiography was immediately performed to assess the right atrial mass. It showed a free-floating thrombus in the right atrium (RA) with a ‘snake-like’ configuration (Fig.1, bottom panel).

 The right ventricle was moderately enlarged with systolic impairment. The inferior vena cava was free of masses. There was moderate pulmonary hypertension with peak pulmonary pressure of 45 mmHg. Serum biochemistry revealed markedly raised troponin and brain-natriuretic peptide levels. The patient was transferred to the intensive care unit where he was intubated and ventilated, and needed extensive support with vasopressors. He also received thrombolysis with reteplase. A repeat echocardiography after thrombolysis showed absence of the previously seen thrombus in the right atrium, which had likely lysed and migrated to the pulmonary vasculature. The patient, however, never recovered from this massive pulmonary vascular insult and remained in the intensive care unit for the next few days. He deteriorated gradually until he passed away.

## DISCUSSION

Free-floating right heart thrombi are uncommon and usually represent clots travelling from the legs to the pulmonary arteries, often referred to as “thrombi-in-transit”. They are seen in 4-18% of patients presenting with acute massive pulmonary embolism.^1^^-^^3^ Despite their frequent occurrence in patients with pulmonary embolism, they are commonly under-diagnosed. The reported case, therefore, underlines the role of echocardiography as a key investigation in this setting.

Echocardiography (transthoracic and transesophageal) has been frequently used to detect and assess the morphology of the right heart thrombi. It is a simple, noninvasive, painless investigation that is widely available and can be performed at the bedside in an intensive care unit. Morphologically, the right heart thrombi are divided into two types, A and B. Type A thrombi have a worm-like shape, are extremely mobile and mostly represent peripheral venous clots which temporarily lodge into the right heart. Type B thrombi are morphologically similar to the left heart thrombi, are less mobile, attach to the right atrial or ventricular wall, have broad-based attachment indicating that these develop within the right heart.^4^ Also, echocardiography can assess right ventricular function and monitor it during and after thrombolysis. Serial echocardiographic examinations are also useful when the clinical status deteriorates because they may demonstrate thrombus that was not detected on the initial examination.

The presence of right heart thrombi in pulmonary embolism predicts worse prognosis, with higher mortality rate. A meta-analysis in 2002^5^ showed mortality rate up to 27%, while De Vrey has recently (2007) reported mortality to be >44%.^6^ This mortality rate is much more compared to pulmonary embolism without right heart thrombi. This underscores the importance of rapidly diagnosing such thrombi and need for prompt treatment.

The thrombi can be easily mistaken for other physiological or pathological structures within the right heart. It must be differentiated from congenital structures^7^such as a chiari network, persistent eustachian or thebesian valves, atrial septal aneurysms, or acquired conditions such as intracardiac tumors and vegetations.^7^ They can easily mimic myxoma on echocardiography. The associated delay in thrombus removal surgery in this case can result in early mortality.^8^ In cases of doubt, transesophageal echocardiography is helpful. It may allow direct visualization of the thrombus in the pulmonary arteries^9^, or even a thrombus entrapped in the patent foramen ovale.

Free-floating thrombi are an extreme therapeutic emergency and any delay to treatment could be lethal.^10^ The options for treatment include anticoagulation with heparin, thrombolysis or surgical removal of the thrombus. However, the most appropriate therapy remained unclear for a long period of time as the reported series were small and the only meta-analysis involved a heterogenous group of patients.^10^ Chartier et al.^3^ reported that there was no significant difference between these therapeutic approaches in terms of in-hospital mortality. However, recent data suggests better outcome with thrombolysis.^5^^,^^11^ The theoretical advantages of thrombolysis are numerous.^12^^,^^13^ It accelerates thrombus lysis and pulmonary reperfusion, reduces pulmonary hypertension, improves right ventricular function and hence improves right and left ventricular output by reducing RV-LV interdependence.^14^ Moreover, it may dissolve the clot at three locations at the same time: the intracardiac thrombus, the pulmonary embolus, and the venous thrombosis. Finally, it is a simple, rapid and widely applicable treatment that can be administered at the bedside. Torbicki^2^ reported that the favourable result after thrombolysis could be related to the shorter delay between the presumed onset of symptoms and hospitalization in patients where pulmonary embolism was associated with mobile clots in the right heart (2.2 versus 4.5 days). Ferrari^15^ showed that after thrombolysis, 50% of the clots disappeared within 2 hours, whereas the remainder disappeared within 12-24 hrs. This delayed disappearance of the thrombi support the decision to defer surgery after thrombolysis until at least 24 hours. Anticoagulation alone can be hazardous as the thrombi may embolize to the already compromised pulmonary circulation. However, Hinton^16^ reported a case of type A right heart thrombus successfully treated with heparin alone. Surgical embolectomy is another option but carries the risk of inherent delay of few hours, general anesthesia, cardiopulmonary bypass, and the inability to remove coexisting pulmonary emboli beyond the central pulmonary arteries. However, thrombi entrapped within the foramen ovale need to be surgically removed at the earliest as they may be deleterious due to unpredictable systemic embolization.^11^ Percutaneous removal of the thrombus using pigtail catheter is a promising possibility with simultaneous direct thrombolysis into the pulmonary artery, but needs more experience.^17^

## CONCLUSION

Free-floating thrombi in the right heart are rare and usually represent travelling clots from the legs to the lungs. They present a therapeutic emergency due to high mortality rate. Echocardiography is an essential investigation that can be performed at bedside to directly visualize the thrombi, assess and monitor RV function, and help in making treatment decisions.
